# Key wheat *GRF* genes constraining wheat tillering of mutant *dmc*

**DOI:** 10.7717/peerj.11235

**Published:** 2021-04-08

**Authors:** Jing Zhang, Junchang Li, Yongjing Ni, Yumei Jiang, Zhixin Jiao, Huijuan Li, Ting Wang, Peipei Zhang, Mengyao Han, Lei Li, Hongjie Liu, Qiaoyun Li, Jishan Niu

**Affiliations:** 1 Henan Agricultural University, National Centre of Engineering and Technological Research for Wheat/National Key Laboratory of Wheat and Maize Crop Science, Zhengzhou, Henan, China; 2 Shangqiu Academy of Agricultural and Forestry Sciences, Shangqiu, Henan, China

**Keywords:** Wheat (*Triticum aestivum* L.), Tillering, Growth regulating factor, Expression profiles, IAA, GA

## Abstract

Tillering is a key agronomy trait for wheat (*Triticum aestivum* L.) production. Previously, we have reported a dwarf-monoculm wheat mutant (*dmc*) obtained from cultivar Guomai 301 (wild type, WT), and found growth regulating factors (GRFs) playing important roles in regulating wheat tillering. This study is to systematically investigate the roles of all the wheat *GRFs* (*T. aestivum GRFs*, *TaGRFs*) in regulating tillering, and screen out the key regulators. A total of 30 *TaGRFs* were identified and their physicochemical properties, gene structures, conserved domains, phylogenetic relationships and tissue expression profiles were analyzed. The expression levels of all the *TaGRFs* were significantly lower in *dmc* than those in WT at early tillering stage, and the abnormal expressions of *TaGRF2-7*(A, B, D), *TaGRF5-7D, TaGRF10-6*(A, B, D) and *TaGRF11-2A* were major causes constraining the tillering of *dmc*. The transcriptions of *TaGRFs* were significantly affected by exogenous indole acetic acid (IAA) and gibberellin acid (GA3) applications, which suggested that *TaGRFs* as well as IAA, GA signaling were involved in controlling wheat tillering. This study provided valuable clues for functional characterization of *GRF* genes in wheat.

## Introduction

Transcription factors are the most important regulators in plants. They are involved in various biological processes such as plant growth and development, metabolism, reproduction and differentiation (Chen & Cao, 2015; Kim & Tsukaya, 2015). Up to now, more than 60 transcription factors have been found in plants (Zhao et al., 2019). Growth-regulating factor (GRF) is a kind of plant-specific transcription factor, which regulates plant growth and development, plant cell size, participates in chloroplast proliferation and abiotic stress response (Chen & Rajewsky, 2007; Omidbakhshfard et al., 2015; Ruan et al., 2018). The most prominent feature of the GRF proteins is that there are two conserved domains in the N-terminal region, namely QLQ (Gln, Leu, Gln) and WRC (Trp, Arg, Cys) domains (Kim & Kende, 2004).

The first *GRF* gene *OsGRF1* was discovered in rice (*Oryza sativa* L.), and it affects stem elongation by regulating gibberellin signaling (Van der Knaap, Kim & Kende, 2000). With the continuous analyses of plant genomic sequences, *GRF* genes have been identified in various plant species, such as Arabidopsis (*Arabidopsis thaliana*) (nine) (Kim, Choi & Kende, 2003), rice (12) (Choi, Kim & Kende, 2004), tomato (*Solanum lycopersicum*) (13) (Yuan et al., 2017), barley (*Hordeum vulgare*) (12) (Xue et al., 2020), soybean (*Glycine max* (Linn.) Merr) (22) (Chen et al., 2019), quinoa (*Chenopodium quinoa* Willd.) (18) (Shi et al., 2019), tea plant (*Camellia sinensis*) (11) (Wang et al., 2019b), and tobacco (*Nicotiana tabacum*) (30) (Dong et al., 2020).

In recent years, many studies have demonstrated that GRFs play a variety of important regulatory roles in plant growth and development (Ma et al., 2017). GRFs participate in the growth and development of root (Bao et al., 2014), stem (Kim & Lee, 2006), leaf (Horiguchi, Kim & Tsukaya, 2005; Liu et al., 2012, 2015) and flower (Liang et al., 2014; Lee et al., 2015), and play an important role in signal transduction and stress response of plants (Liu et al., 2008; Hewezi et al., 2012; Casadevall et al., 2013). *OsGRF4* controls grain shape, panicle length and seed shattering in rice (Sun et al., 2016). However, there is almost no evidence of the *GRFs* regulating tiller development in wheat. A recent study indicates that *TaGRFs* may be involved in regulating wheat (*Triticum aestivum* L.) tillering (Wang et al., 2019a).

Common wheat is one of the most important food crops worldwide. Previously, we have reported a dwarf-monoculm wheat mutant (*dmc*) derived from Guomai 301 (He et al., 2018), and found that wheat miR396b (tae-miR396b) and several of its target *TaGRFs* are involved in regulating wheat tillering in *dmc* (An et al., 2019). This provides a good opportunity to design experiments exploring the molecular mechanisms of wheat tiller development. Here, we attempt to thoroughly investigate the expression profiles of all *TaGRFs* in Guomai 301 and mutant *dmc* under normal growth and development condition, as well as exogenous indole acetic acid (IAA) and gibberellin acid (GA) applications. The results provided a theoretical base for further functional characterization of *GRF* genes in wheat.

## Materials and Methods

### Plant materials and growth conditions

The mutant *dmc* was obtained according to the method described in the previous article (An et al., 2019). All the plant materials were planted in our experimental field (34°25′ N, 115°39′ E, 49 m *a.s.l*.). The field experiments were carried out in a completely randomized design as described by Li et al. (2019). Fertilizer and weed management were similar to that of wheat breeding (Li et al., 2014).

### IAA and GA treatments

For hormone treatments, the wheat seedlings at the early three-leaf stage were sprayed with 10^−5^ mol L^−1^ IAA solution, and the wheat seedlings at the three-leaf stage were sprayed with 2 × 10^−4^ mol L^−1^ GA solution on the leaves until all the leaves were wet. The tillers of seedlings were sampled at 0 (untreated control), 1 and 2 h after hormone treatments. The RNAs of all the samples were immediately extracted.

### Tiller sample preparation

Three bulks of tiller samples were prepared separately at the three-leaf stage (WT1, *dmc*1; sampling date: 15 November 2018), the over-winter stage (WT2, *dmc*2; sampling date: 6 January 2019) and the rising to jointing stage (WT3, *dmc*3; sampling date: 16 February 2019) for RNA extraction. The RNAs were used for qRT-PCR analysis. Wheat tillering had been completed at the rising to jointing stage. All the samples had three biological replicates.

### Identification of *TaGRFs*

The genome sequences and protein sequences of latest wheat genome assembly version IWGSC refseqv1.1 (http://plants.ensembl.org/) was used to identify wheat GRF family members. The longest transcript sequence corresponding to each candidate gene was selected as the final sequence.

The Hidden Markov Model (HMM) of WRC (PF08879) and QLQ (PF08880) domains were obtained from the Pfam website (http://pfam.xfam.org/) and were used to identify the conserved domains of wheat GRF proteins with HMMER software (https://www.ebi.ac.uk/Tools/hmmer/). Besides, the protein sequences of Arabidopsis GRF family members and rice GRF family members were downloaded from PlantTFDB (http://planttfdb.cbi.pku.edu.cn/), and these sequences were used as input sequences to BLAST in the wheat protein database. All output protein sequences with *e*-value ≤ 1 × 10^−10^ were collected, removing the redundant sequences. The domains of the candidate GRF family members were confirmed by searching Pfam website and the Conserved Domain Search at NCBI (https://www.ncbi.nlm.nih.gov/Structure/bwrpsb/bwrpsb.cgi). The *TaGRF* genes were named mainly to refer to the annotation information from the UniProt database (https://www.uniprot.org/) and their chromosomal locations.

The lengths of amino acid sequences, molecular weights, pIs and other characteristics of wheat GRF proteins were analyzed using the online analysis software of EXPASY website (https://www.expasy.org/). All identified *TaGRF* genes were located on chromosomes, and the gene map was drawn using TBtools software (Chen et al., 2020).

### Construction of the phylogenetic tree

Amino acid sequences of the GRF proteins in wheat, Arabidopsis and rice were used to conduct multi-sequence alignment. Based on the results of sequence alignment, the phylogenetic tree was constructed using the Neighbor-Joining method in MEGA software (https://www.megasoftware.net/). The check parameter bootstrap value was 1,000, and the default value of the system was used for other parameters.

### Conserved motif and gene structure analysis of *TaGRFs*

The online website MEME (http://meme-suite.org/) was used to analyze the motifs of TaGRFs protein sequences. Parameters were set as follows: the motif discovery mode was classic mode, the site distribution was Zero or One Occurrence Per Sequence (zoops), the maximum number of motifs to find was 10, and other parameters were default. The DNA and cDNA sequences corresponding to predicted proteins from the wheat genome database were downloaded from the wheat genome database. Then, TBtools software was used to draw motif distribution and the structure distribution map of TaGRFs, and the Logo diagram of amino acid conservation was drawn on the WEBLOGO website.

### Analysis of the *cis*-acting elements in *TaGRF* promoters

The 2,000 bp upstream sequences before transcription start positions of *TaGRFs* were extracted from wheat genome sequence, and the *cis*-acting elements were analyzed using the PlantCARE database (http://bioinformatics.psb.ugent.be/webtools/plantcare/html).

### Gene duplication and synteny analysis of *TaGRFs*

Multiple Collinearity Scan toolkit (MCScanX) program with the default parameters was adopted to analyze the gene duplication events of *TaGRFs*. To exhibit the synteny relationship of the orthologous *GRF* genes obtained from wheat and other selected species, the syntenic analysis maps were constructed using the Dual Synteny Plotter software (https://github.com/CJ-Chen/TBtools). According to the results of MCScanX, the nonsynonymous substitution rate (Ka) and synonymous substitution rate (Ks) of duplicated *GRF* genes were calculated by KaKs Calculator 2.0 software. The Ka/Ks ratios for *TaGRF* genes were used to assess the selection pressure on duplicated genes and Ka/Ks ratio >1, <1, or =1 indicated positive, negative, or neutral evolution, respectively.

### Tissue specific expression analysis of *TaGRFs*

The raw gene expression data were downloaded from the Wheat Expression Browser (http://www.wheat-expression.com/) (Table S1). A total of eight mRNA-seq data from wheat cultivar Chinese Spring and Azhurnaya were analyzed. The samples were prepared from four tissues, including spike (anthesis stage), grain (milk grain stage), leaves/shoots (flag leaf stage) and roots (flag leaf stage). TBtools software was used to draw the expression heat map of *TaGRFs* in wheat. The expression profiles were performed based on the transcripts per million (TPM) values of *TaGRF* genes. The Gene Ontology database (GO, geneontology.org) was used for functional annotation of the *GRF* genes. GO annotations were mapped according to molecular functions, biological processes and cellular components (Table S2).

### RNA-seq and gene expression analysis

The samples for RNA-seq analysis were prepared at the three-leaf stage to four-leaf stage in 2017. Two super bulk samples of the mutant *dmc* (T01, T02 and T03) and WT (T04, T05 and T06) with three biological replicates were prepared. The transcript abundance of *TaGRF* genes was calculated as fragments per kilobase of exon model per million mapped reads (FPKM) (He et al., 2018). The expression profiles of all the *TaGRF* genes in Guomai 301 and mutant *dmc* were analyzed based on the RNA-seq data in this study (Table S3). The heatmaps were drawn by HemI1.0 based on the transformed data of log_2_ (FPKM + 1) values (Deng et al., 2014).

### qRT-PCR

qRT-PCR was performed as described previously by An et al. (2019). The primers of *TaGRFs* were designed using Primer-Blast of NCBI website (https://www.ncbi.nlm.nih.gov/tools/primer-blast/). All the primer sequences were listed in Table S4. The β-actin gene was used as an internal control and each reaction was performed with triplicates. The relative expression of *TaGRFs* was calculated by 2^−ΔΔCt^ methods (Livak & Schmittgen, 2001).

## Results

### Typical traits of Guomai 301 and mutant *dmc*

**Figure 1:**
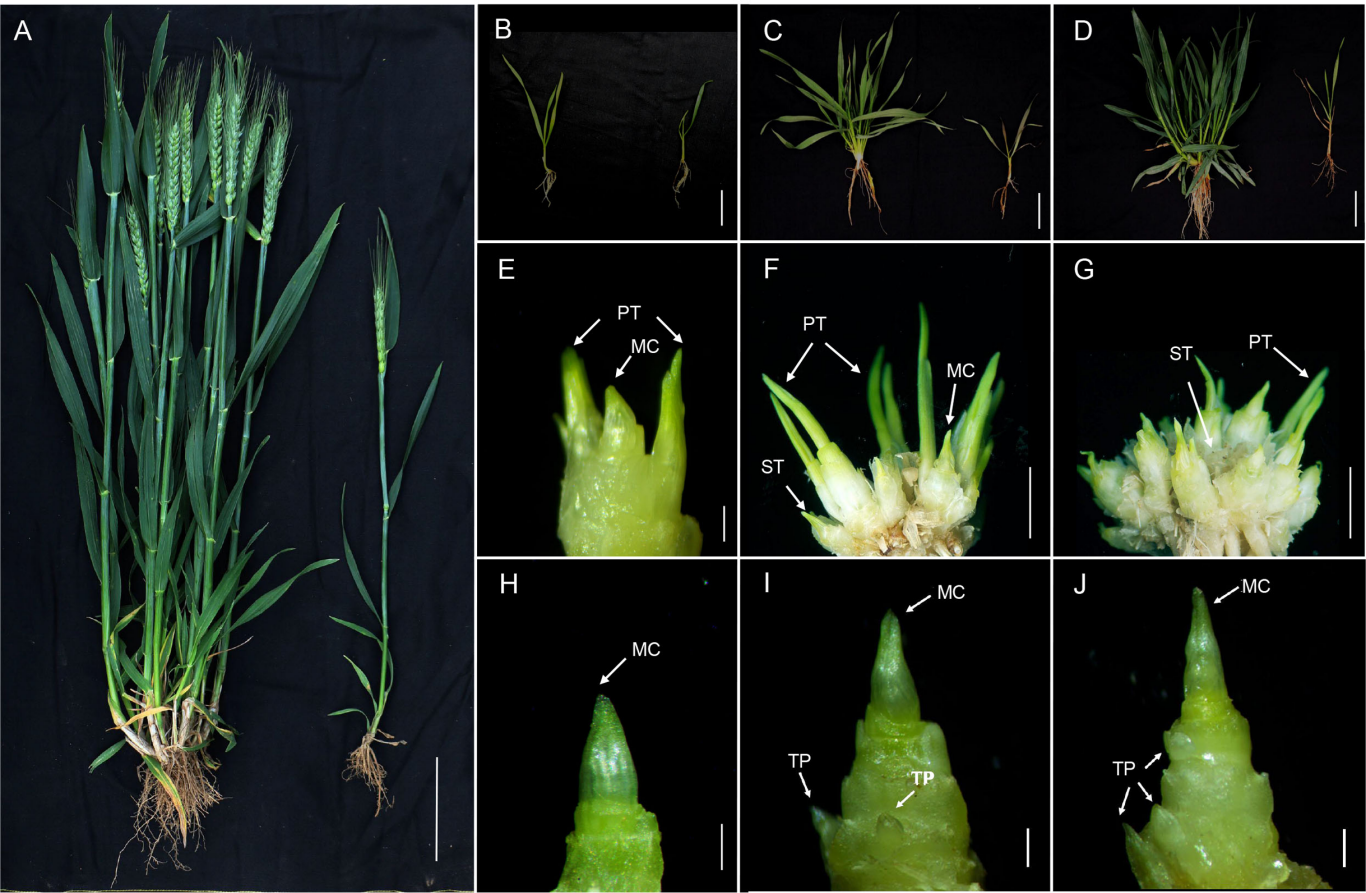
The tiller micromorphology of Guomai 301 and mutant *dmc*. (A) The plant phenotype of Guomai 301 (left) and mutant *dmc* (right). (B) Guomai 301 (left) and *dmc* (right) at the three-leaf stage. (C) Guomai 301 (left) and *dmc* (right) at the over-winter stage.(D) Guomai 301 (left) and *dmc* (right) at the rising to jointing stage. (E–G) WT at the three-leaf stage, over-winter stage, and the rising to jointing stage. (H–J) *dmc* at the three-leaf stage, over-winter stage, and the rising to jointing stage. MC, main culm; TP, tiller primordium; PT, primary tiller; ST, secondary tiller. (A) Scale bar = 10 cm; (B)–(D) Scale bar = 2 cm; (E) and (H)–(J) scale bar = 1 mm; (F) and (G) Scale bar = 1 cm.

The plant height and tiller number between mutant *dmc* and Guomai 301 were significantly different at heading stage (Fig. 1A). The average plant height and tiller number of Guomai 301 were 64.9 cm and 20.33, the average plant height of mutant *dmc* was 48.0 cm and most *dmc* plants had no tiller. At the three-leaf stage (Fig. 1B), the tiller buds of WT formed two primary tillers (PTs) at the base of the main culm (Fig. 1E). Meanwhile, only one tiny protuberance formed at the main culm base of *dmc* (Fig. 1H). At the over-winter stage (Fig. 1C), the tiller number of WT had reached 11–14, which mainly consisted of PTs and secondary tillers (Fig. 1F), while there were only two tiny tiller primordia (TPs) at the base of the *dmc* (Fig. 1I). Between the rising stage and the jointing stage (Fig. 1D), the tiny TPs of *dmc* were almost unchanged as before (Fig. 1J); but the tiller number of WT had reached its maximum value (Fig. 1G) (An et al., 2019).

### Identification of *TaGRFs*

A total of 30 *TaGRF* genes (including 13 homoeologous groups) (Table 1) were finally identified from the wheat genome using nine Arabidopsis *GRF* and 12 rice *GRF* genes as input sequences. The lengths of TaGRF proteins ranged from 211 to 611 aa and the mean value was 359 aa; the molecular weight ranged from 22.3 to 64.3 kDa and the average value was 38.8 kDa. The TaGRF6-4B was the heaviest and TaGRF10-6B was the lightest. The isoelectric point (pI) was between 4.72 and 9.90, with an average value of 7.9, and the TaGRF10-6D was the highest and TaGRF11-2B was the lowest. Among them, seven TaGRFs with pIs less than seven were slightly acidic, while the remaining 23 TaGRFs with pIs greater than seven were slightly alkaline. These data indicated that most TaGRFs contained more basic amino acids. The fat solubility of TaGRFs was between 45.83 and 70.6, with an average value of 57.056. Their hydrophobic indices ranged from −0.882 to −0.35, indicating that TaGRFs had good hydrophilicity (Grand average of hydropathicity, GRAVY).

**Table 1:** Some basic characteristics of wheat *GRF* genes.

Gene name	Gene ID	Physical position and gene direction	Protein length (aa)	Isoelectric point (pI)	Molecular weight (Da)	Aliphatic index	GRAVY
*TaGRF1-6A*	TraesCS6A02G335900.1	568515906–568517951	409	7.22	44,786.45	46.65	−0.833
*TaGRF1-6B*	TraesCS6B02G366700.1	639532839–639534932	410	7.21	44,724.4	45.83	−0.821
*TaGRF1-6D*	TraesCS6D02G315700.1	423814859–423816885	414	7.24	45,263.95	46.09	−0.835
*TaGRF2-6A*	TraesCS6A02G174800.1	188250927–188252018	315	8.12	33,604.85	70.6	−0.35
*TaGRF2-7A*	TraesCS7A02G165600.1	121056090–121057606	309	8.55	34,214.05	51.33	−0.841
*TaGRF2-7B*	TraesCS7B02G070200.1	76903837–76905287	316	8.55	34,886.68	49.59	−0.882
*TaGRF2-7D*	TraesCS7D02G166400.1	117132680–117134270	320	8.26	35,405.24	50.5	−0.882
*TaGRF3-2A*	TraesCS2A02G435100.1	687048698–687052485	384	6.76	42,326.85	54.61	−0.623
*TaGRF3-2D*	TraesCS2D02G435200.1	546208318–546212200	391	7.04	42,780.33	53.4	−0.62
*TaGRF4-2B*	TraesCS2B02G458400.1	653016354–653019980	387	7.01	42,454.97	52.4	−0.621
*TaGRF4-6A*	TraesCS6A02G269600.1	496010434–496017229	408	7.65	43,457.29	52.03	−0.579
*TaGRF4-6B*	TraesCS6B02G296900.1	532899954–532903804	406	8.46	43,435.28	53.03	−0.571
*TaGRF4-6D*	TraesCS6D02G245300.1	347433246–347436961	409	8.16	43,620.48	52.4	−0.559
*TaGRF5-4A*	TraesCS4A02G434900.1	705509912–705513259	371	8.5	39,941.33	47.44	−0.726
*TaGRF5-7A*	TraesCS7A02G049100.1	22937762–22940558	370	8.78	40,161.6	46.24	−0.762
*TaGRF5-7D*	TraesCS7D02G044200.1	22586526–22589173	368	8.57	39,895.24	46.77	−0.75
*TaGRF6-4A*	TraesCS4A02G255000.1	567181384–567185511	607	6.87	63,953.28	65.58	−0.416
*TaGRF6-4B*	TraesCS4B02G060000.1	51679625–51683720	611	6.72	64,277.53	65.01	−0.423
*TaGRF6-4D*	TraesCS4D02G059600.1	35476875–35481038	578	6.58	61,162.1	64.43	−0.45
*TaGRF9-4A*	TraesCS4A02G291500.1	594532829–594535533	408	9	45,327.7	55.78	−0.842
*TaGRF9-4D*	TraesCS4D02G020300.1	8777205–8779937	415	8.82	45,994.94	56.75	−0.794
*TaGRF10-6A*	TraesCS6A02G257600.1	479833651–479834702	212	9.54	22,597.56	67.36	−0.384
*TaGRF10-6B*	TraesCS6B02G267500.1	481089083–481090143	211	9.64	22,347.22	67.68	−0.382
*TaGRF10-6D*	TraesCS6D02G238900.1	339389350–339390413	215	9.9	22,750.77	68.74	−0.36
*TaGRF11-2A*	TraesCS2A02G238700.1	323528641–323530709	319	4.89	34,683.79	66.68	−0.492
*TaGRF11-2B*	TraesCS2B02G256600.2	298324714–298327379	263	4.72	28,160.15	61.63	−0.689
*TaGRF11-2D*	TraesCS2D02G246600.1	288225741–288228187	264	4.76	28,182.2	62.16	−0.692
*TaGRF12-2A*	TraesCS2A02G398300.2	651752444–651753590	225	9.82	23,823.85	64.36	−0.436
*TaGRF12-2B*	TraesCS2B02G416300.1	594982278–594983776	227	9.82	24,077.17	62.51	−0.479
*TaGRF12-2D*	TraesCS2D02G395900.1	506941472–506942539	229	9.57	24,221.3	64.1	−0.443

**Figure 2:**
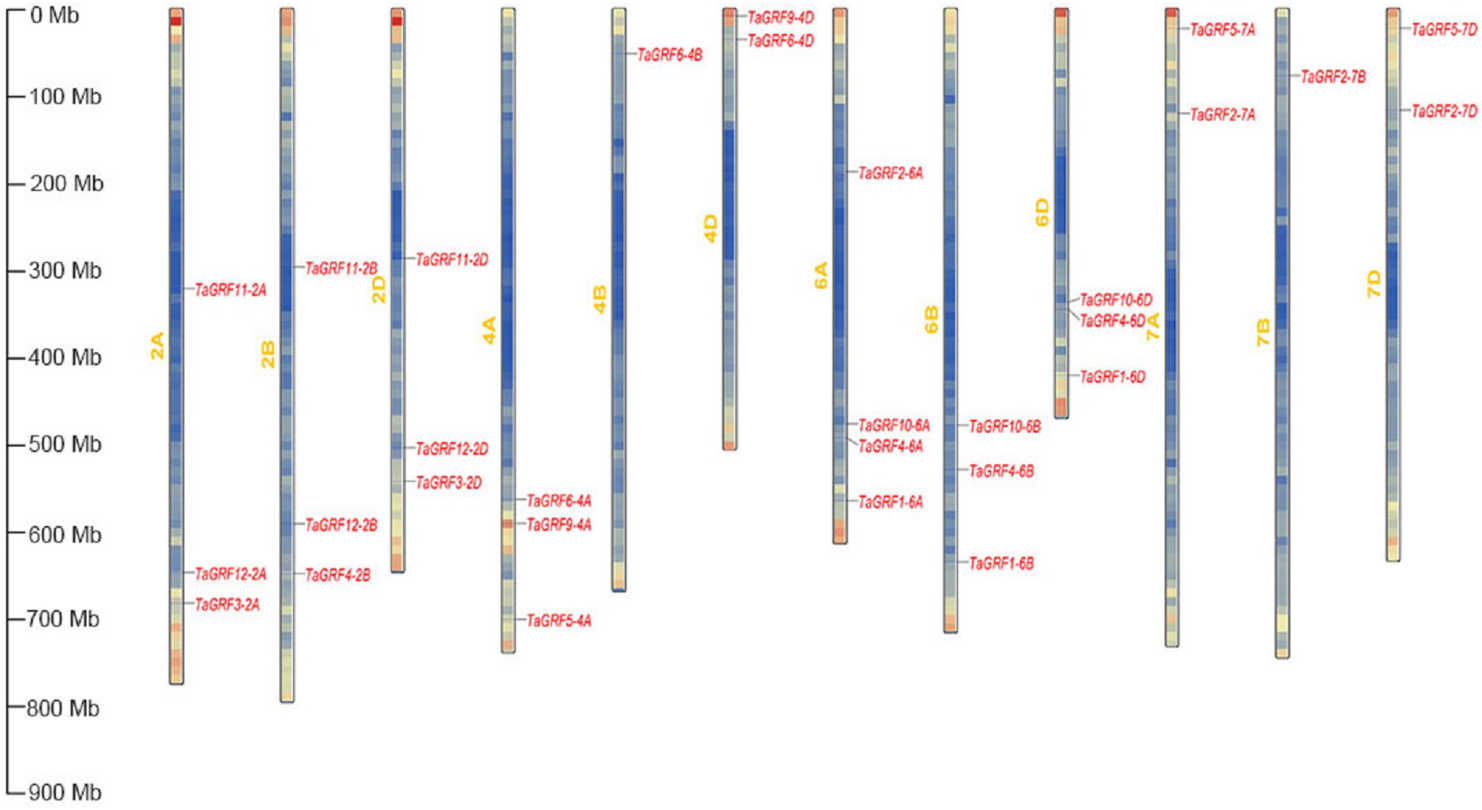
The chromosomal distributions of *TaGRF* genes. The density of genes on each chromosome is uneven, red and blue denote higher and lower gene density, respectively.

The 30 *TaGRFs* were located on 12 of the 21 wheat chromosomes. Each of the 12 chromosomes had 1–4 *TaGRFs*. Among them, chromosome 6A had four *TaGRFs*, 4B and 7B had one *TaGRF* and 4D, 7A and 7D had two *TaGRFs*, respectively (Fig. 2).

### Phylogenetic tree of wheat GRF proteins

**Figure 3:**
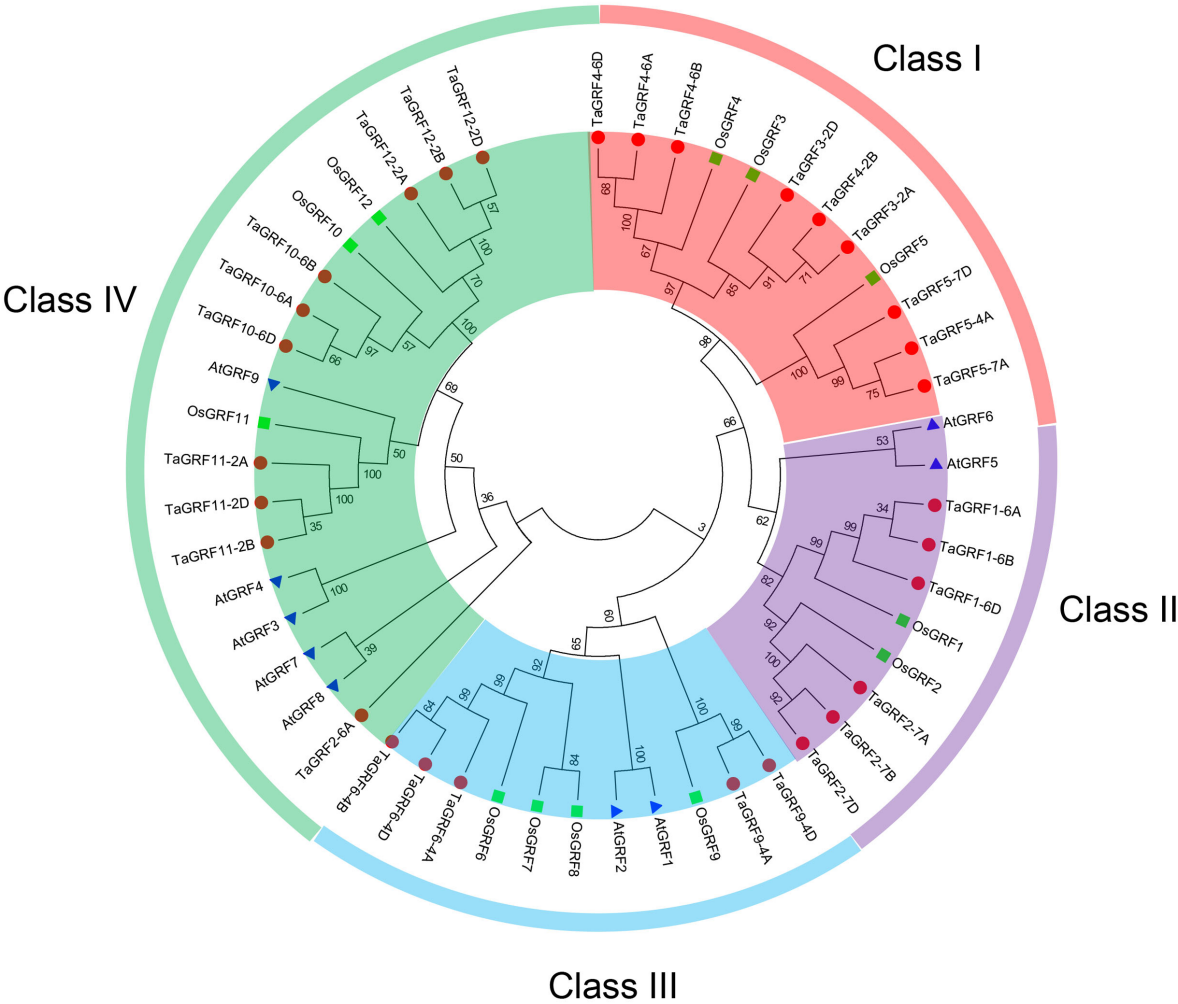
The phylogenetic tree of the GRFs in *T. aestivum* L. (Ta), *A. thaliana* (At), and *O. sativa* (Os).

In order to better understand the phylogenetic relationship between TaGRFs and those in other plant species. The phylogenetic tree was constructed (Fig. 3), including 30 TaGRFs, nine AtGRFs and 12 OsGRFs. According to the branches of the phylogenetic tree, the 51 GRFs were clustered into four classes: Class I, II, III and IV. There were nine TaGRFs in Class I, six TaGRFs in Class II, five TaGRFs in Class III and 10 TaGRFs in Class IV. Phylogenetic analysis indicated that wheat GRFs were more closely related to rice GRFs than Arabidopsis GRFs.

### Structures and conserved motifs of wheat *GRF* genes

**Figure 4:**
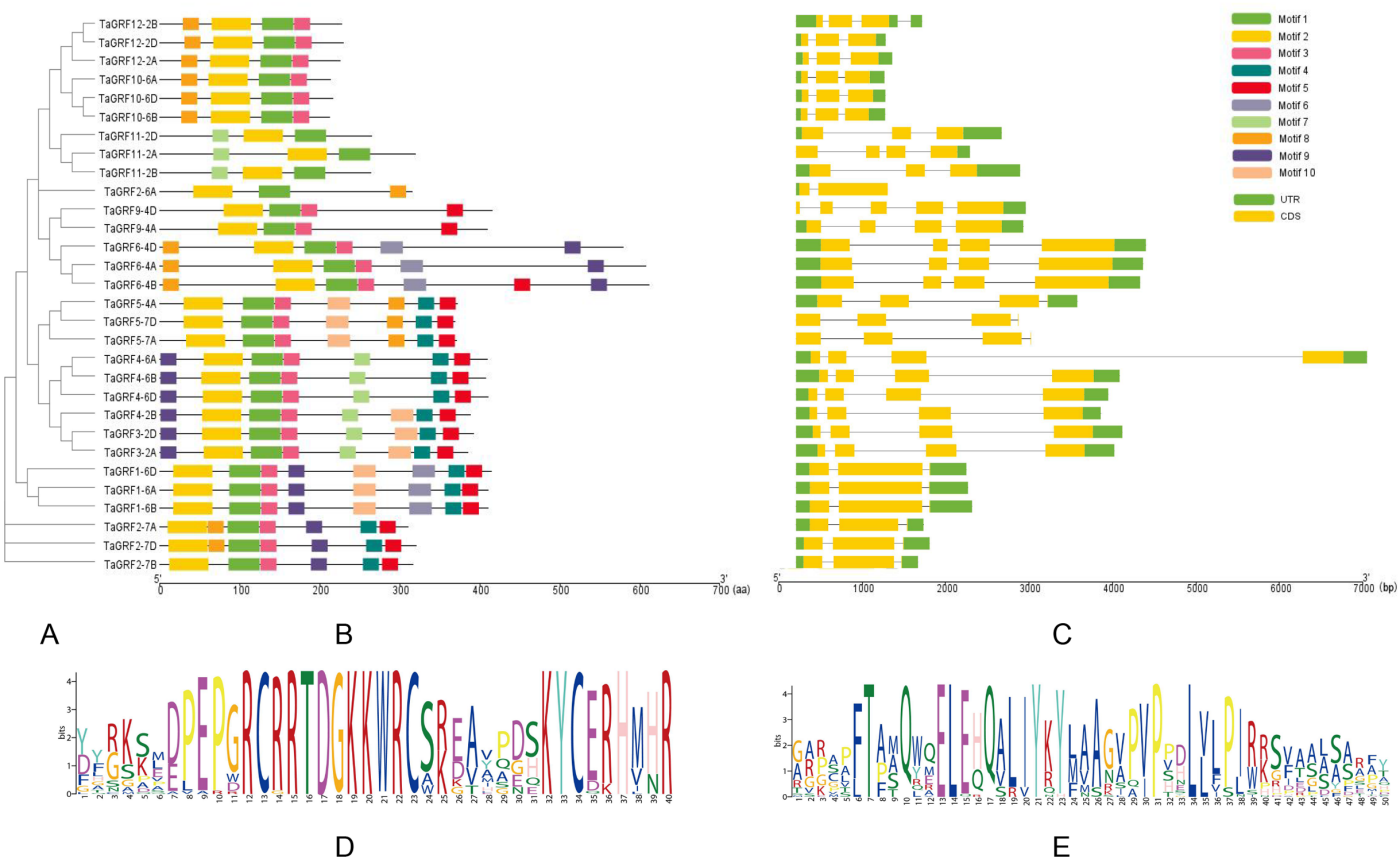
Phylogenetic relationships, gene and protein structures of *TaGRFs*. (A) The phylogenetic tree was constructed based on the full-length sequences of wheat GRF proteins using MEGA software. (B) The motif compositions of TaGRFs. The motifs, numbers 1–10, are displayed in different colored boxes. (C) Exon-intron structures of *TaGRFs*. Green boxes indicate untranslated 5′- and 3′-regions; yellow boxes indicate exons; black lines indicate introns. (D and E) The conserved sequences of motif 1 (left) and motif 2 (right) in TaGRFs. The lengths of the proteins (B, aa) and DNAs (C, bp) can be estimated using the scales at the bottoms.

Based on the gene structural information, the phylogenetic tree of *TaGRFs* was built (Fig. 4A). A total of 10 conservative motifs were predicted in wheat GRF proteins (Fig. 4B). Motif 1 was the WRC domain and motif 2 was the QLQ domain, both were highly conserved and existed in the protein sequences of all wheat GRF proteins (Figs. 4D and 4E). In addition, most *TaGRFs* also contained motif 3. The exon-intron structure diagram of *TaGRFs* showed that their genomic DNA sequence lengths were significantly different (Fig. 4C). The longest was *TaGRF4-6A*, its length was about 7,000 bp, and the shortest was about 1,000 bp. The exon number of *TaGRFs* was 2–5, and the exon numbers of *TaGRFs* were closely related to their classes in the phylogenetic tree of TaGRFs (Fig. 3). For example, each of the six *TaGRFs* in Class II had two exons. Among the 30 *TaGRFs*, 25 *TaGRFs* had both 5′-UTR (untranslated region) and 3′-UTR, two *TaGRFs* only contained 3′-UTR, one *TaGRF* only contained 5′-UTR, and the remaining two *TaGRFs* had no UTRs.

### *Cis*-acting elements in the promoters of *TaGRFs* and GO annotation

**Figure 5:**
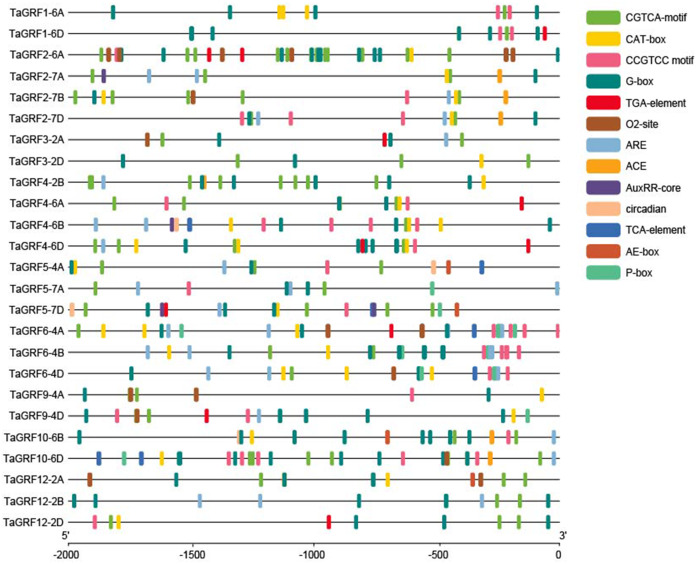
The *Cis*-acting elements in the promoters of *TaGRFs*.

Among the members of the wheat *GRF* gene family, the promoter sequences of *TaGRF1-6B*, *TaGRF10-6A*, *TaGRF11-2A*, *TaGRF11-2B* and *TaGRF11-2D* contained a large number of ‘N’, so they hadn’t been analyzed (Fig. 5). Except for a large number of CAAT-box and TATA-box elements, there were also a large number of *cis*-acting regulatory elements related to growth and development, hormones and stress responsiveness in the promoter sequences of *TaGRFs*, including some *cis*-acting elements involving in auxin (AuxRR-core, TGA-element), gibberellin (P-box), light response (ACE, AEbox and G-box), methyl jasmonate reaction (CGTCA-motif), salicylic acid reaction (TCA-element), circadian rhythm control (circadian), regulation of gliadin metabolism (O2-site), and regulation of meristematic expression (CAT-box and CCGTCC motif). These *cis*-acting elements implied *TaGRFs* played various potential roles in regulating wheat growth and development, and response to hormones and stresses.

Gene Ontology annotation revealed that the *TaGRF* genes are mainly involved in regulation of transcription, DNA-templated (GO:0006355) and response to deep water (GO:0030912). Both *TaGRF1* and *TaGRF2* are involved in response to gibberellin (GO:0009739) (Table S2).

### Synteny analysis of *TaGRFs*

**Figure 6:**
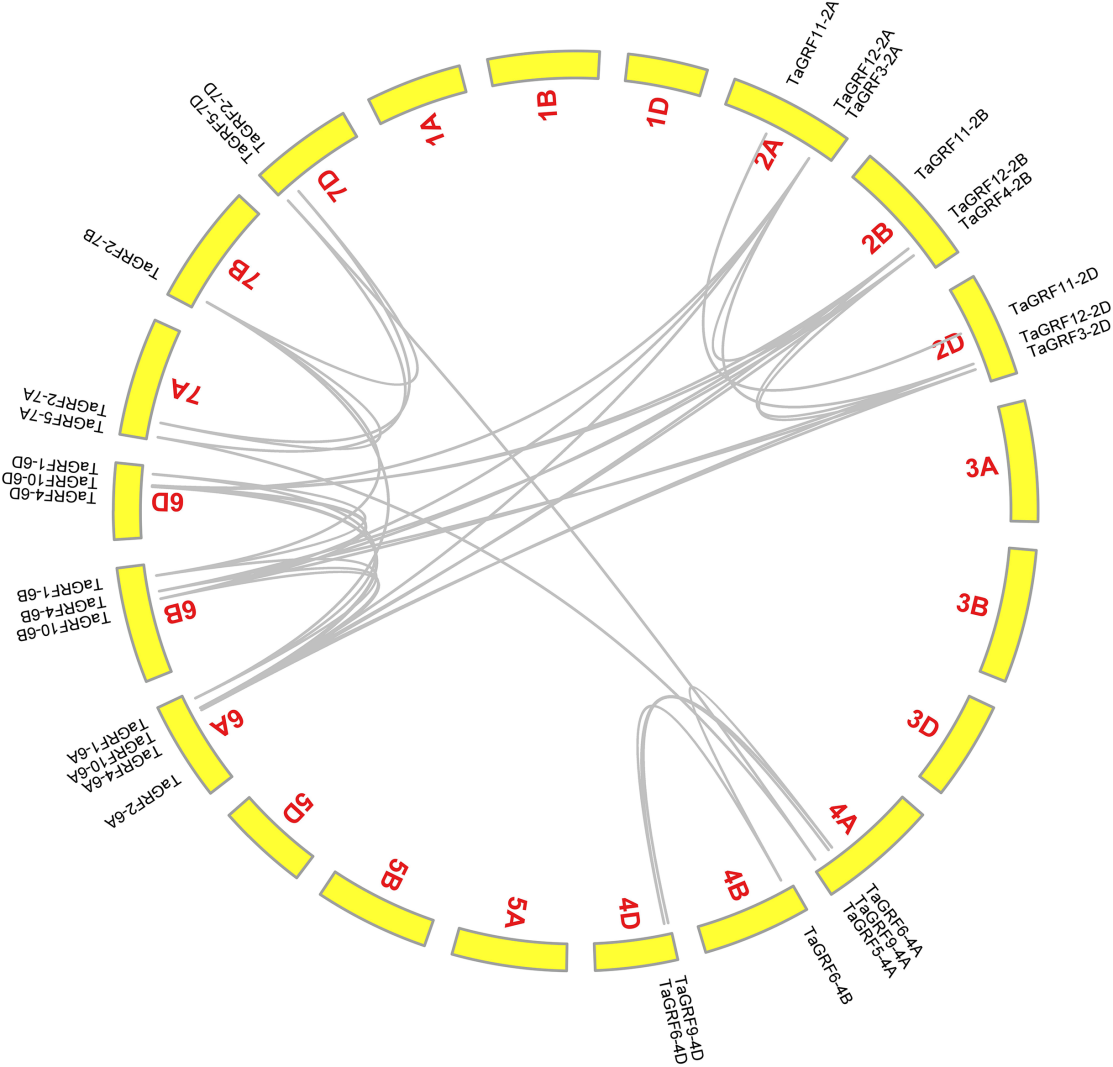
Schematic diagram of the chromosome distribution and interchromosome relationships of *TaGRFs*. The grey lines indicate probably duplicated *TaGRF* gene pairs.

A total of 39 *TaGRF* gene pairs were probably segmental duplicated genes, including 21 pairs (24 genes) of homoeologous genes, and they distributed on different chromosomes (Fig. 6; Table S5). According to the methodology of Holub (2001), there were no tandem duplication genes in wheat *GRF* gene family, and most *TaGRFs* were generated by large-scale repeated events or segmental duplications. It implied that the segmental duplication played an important role in the evolution of the *TaGRF*s.

**Figure 7:**
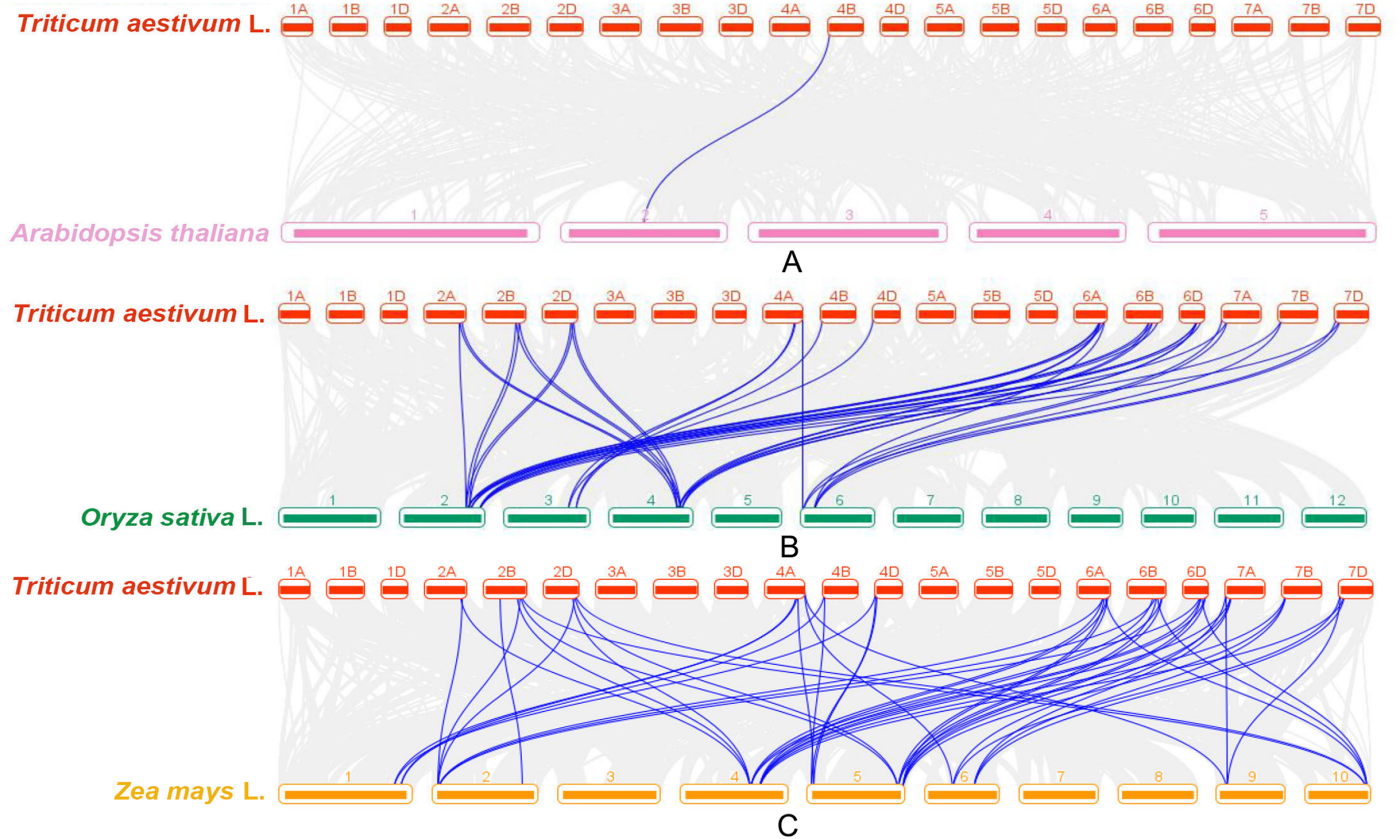
Synteny analyses of *GRFs* between wheat and three representative plant species. (A) The synteny analyses of *GRFs* between wheat and Arabidopsis. (B) The synteny analyses of *GRFs* between wheat and rice. (C) The synteny analyses of *GRFs* between wheat and maize. Gray lines in the background indicate all collinear blocks within wheat and other plant genomes, while the blue lines highlight the syntenic *GRF* gene pairs.

Three comparative syntenic maps of wheat were constructed compared with three representative plant species, including Arabidopsis, rice and maize (Fig. 7). Only one wheat *GRF* gene, *TaGRF6-4B*, had a syntenic gene in Arabidopsis (*AtGRF1*) (Fig. 7A). The numbers of syntenic gene pairs between wheat and rice (Fig. 7B), wheat and maize (Fig. 7C) were 40 and 51 (Table S6). It indicated that the evolutions of *GRF* genes in wheat, rice and maize were similar.

To better understand the evolutionary constraints acting on *TaGRFs*, we calculated the Ka/Ks ratios of these syntenic gene pairs. The results showed that the Ka/Ks ratios of most *TaGRF* syntenic gene pairs were less than 1 (Table S7), suggesting that the *TaGRFs* might have undergone purifying selection processes.

### The expression patterns of *TaGRFs* in different tissues

**Figure 8:**
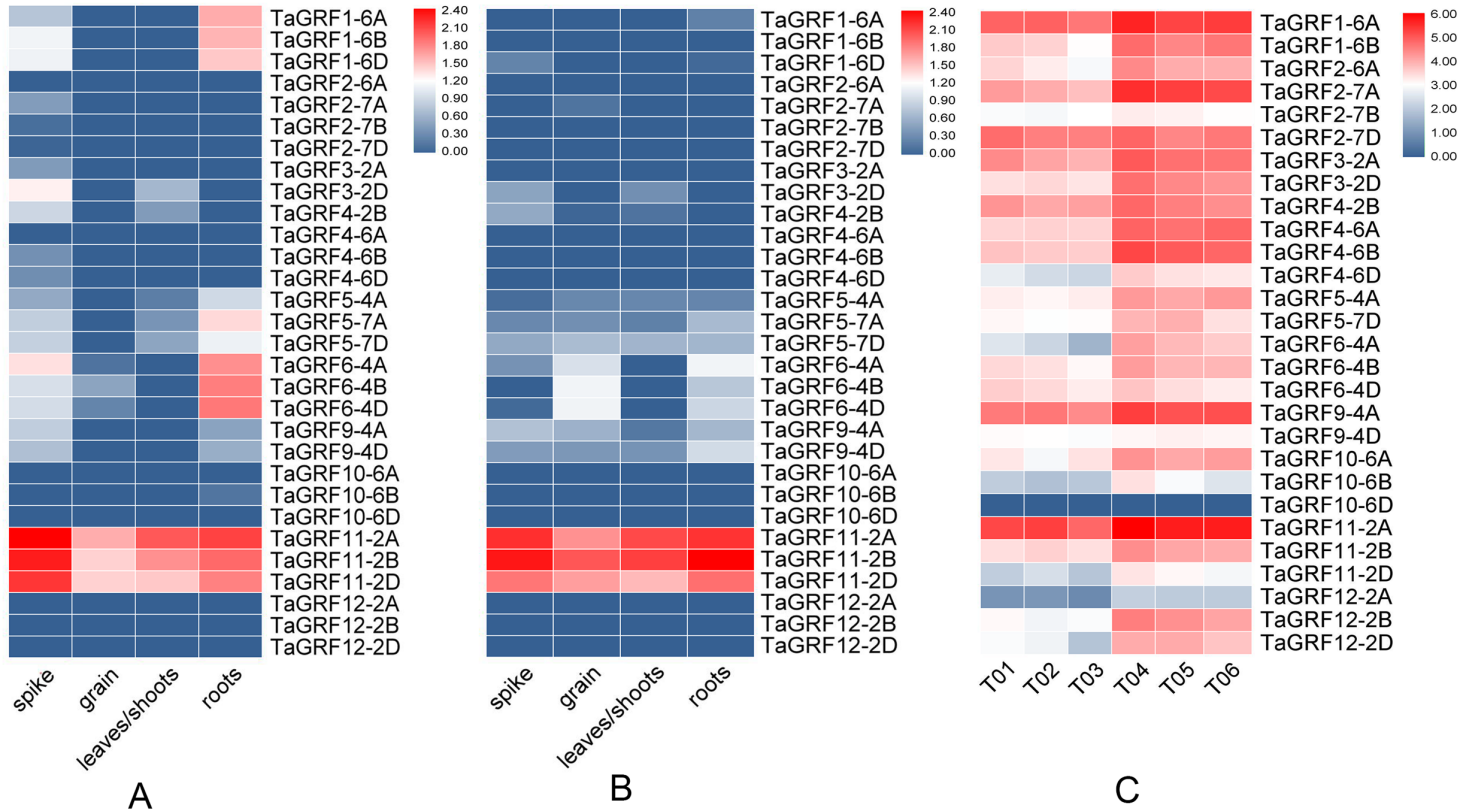
Expression profiles of the *TaGRF* genes in different wheat tissues. (A) Heatmap of expression profiles of *TaGRFs* in Chinese Spring (CS). (B) Heatmap of expression profiles of *TaGRFs* in Azhurnaya. (C) Heatmap of *TaGRF* genes in tiller primordia of WT and *dmc* based on transcriptome data. Three biological replicates were set up in the mutant *dmc* (T01, T02 and T03) and WT (T04, T05 and T06), and each sample bulk of tiller primordia included more than 10 independent individuals. Note: Blue: Low expression level; Red: High expression level; The gene expression values present as log_2_-transformed normalized TPM values.

The expression profiles of *TaGRFs* in spike, grain, leaf/shoot and root showed that about half of 30 *TaGRFs* had similar expression patterns in Chinese Spring and Azhurnaya, and the others expressed differentially in the four tissues of Chinese Spring (Fig. 8A). The expression patterns of most *TaGRFs* in Azhurnaya were similar to that in Chinese Spring. However, the expression profiles of *TaGRF1*, *TaGRF6* and *TaGRF9* were significantly different between Azhurnaya and Chinese Spring (Fig. 8B). The expression levels of all the three homoeologous genes of *TaGRF11-2*(A, B, D) in the four tissues were high, while the expressions of *TaGRF2*, *TaGRF4*, *TaGRF10* and *TaGRF12* were not detected or their levels were very low, implying they played no important roles in the development of the four tissues. *TaGRF1*, *TaGRF5*, *TaGRF6* and *TaGRF9* expressed highly in spike and root, implying their roles in regulating spike and root development. Tissue-specific expression profiles of some *TaGRFs* between Azhurnaya and Chinese Spring were slightly different, which was probably caused by genotype, sample, and experiment variations. These results can be used as a reference for functional studies of *TaGRFs*.

### Expressions of *TaGRFs* in TPs of the mutant *dmc*

The expression patterns of all 30 *TaGRFs* were investigated using available transcriptome data (Fig. 8C). Among the 30 *TaGRFs*, *TaGRF1-6D* and *TaGRF5-7A* had not been detected, *TaGRF10-6D* was almost not expressed, and *TaGRF12-2A* expressed very lowly in all detected samples, these four genes probably were not necessary for wheat tiller development (Fig. 8C). *TaGRF1-6A* (FPKM > 20 in *dmc*), *TaGRF9-4A* and *TaGRF11-2A* (FPKM > 24 in *dmc*) expressed relatively highly indicated their basic functions in wheat tiller development. All *TaGRFs* expressed at a reduced level in *dmc* than in WT. Compared to WT, the expression level of *TaGRF6-4A* decreased by 66.2%, *TaGRF12-2B* decreased by 57.8%, and other 15 *TaGRFs* decreased by more than 40% in *dmc*. This indicated that the expressions of all the *TaGRFs* played positive roles at early tillering stage, the constrained tillering of the *dmc* was associated with the lower expression levels of *TaGRFs*.

**Figure 9:**
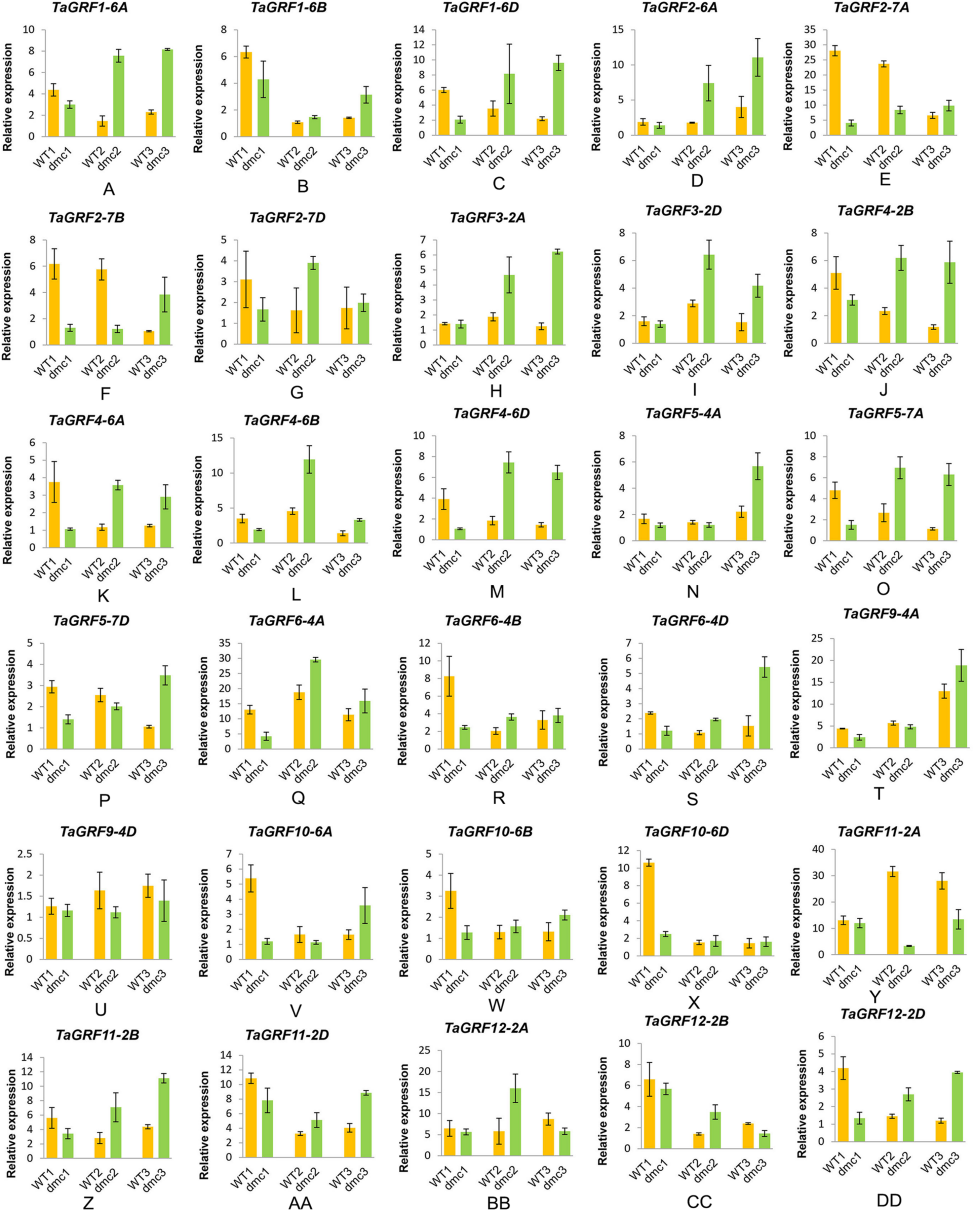
Expression analysis of 30 *TaGRF* genes at three tillering stages by qRT-PCR. (A–DD) The qRT-PCR results of 30 *TaGRF* genes in the tiller primordia of WT and *dmc* at three tillering stages. WT1, *dmc*1: the three-leaf stage; WT2, *dmc*2: the over-winter stage; WT3, *dmc*3: the rising to jointing stage. Data were normalized to β-actin gene and vertical bars indicated standard deviation.

qRT-PCR was performed to analyze the expression patterns of *TaGRFs* in the TPs of WT and mutant *dmc* at three tiller developmental stages (Fig. 9). The 30 *TaGRF* genes had various expression patterns at three tillering stages. All the 30 *TaGRFs* expressed at a reduced level in *dmc* at the three-leaf stage, which was consistent with the transcriptome data. A total of 20 *TaGRFs* expressed highly in *dmc* at the over-winter stage and the rising to jointing stage, while *TaGRF12-2A* and *TaGRF12-2B* expressed lowly in *dmc* at the rising to jointing stage. Among these genes, *TaGRF1-6D, TaGRF2-7D, TaGRF4-6A, TaGRF4-6D, TaGRF5-7A, TaGRF6-4B, TaGRF6-4D* and *TaGRF12-2D* expressed significantly differentially in *dmc* and WT at wheat tillering stages, implying their important roles in the abnormal tillering in *dmc*. The expression levels of *TaGRF2-7A, TaGRF2-7B, TaGRF5-4A, TaGRF5-7D, TaGRF9-4A, TaGRF9-4D, TaGRF10-6A* and *TaGRF11-2A* were always lower in *dmc* at two tillering stages, the expression levels of *TaGRF2-7A, TaGRF2-7B* and *TaGRF11-2A* in *dmc* were significantly different from that in WT. *TaGRF9-4D* and *TaGRF11-2A* expressed lowly in *dmc* compared to that in WT at each stage, implying they might affect the tillering of *dmc*. There were 15 *TaGRFs* expressed at an upward trend in *dmc. TaGRF2-6A, TaGRF9-4A* and *TaGRF9-4D* expressed at an upward trend in WT, implying their roles in regulating tiller development. *TaGRF10-6D* and *TaGRF12-2B* expressed at a downward trend in *dmc*. The expression patterns of *TaGRF* genes were complex. In summary, the abnormal expressions of *TaGRF2-7*(A, B, D), *TaGRF5-7D, TaGRF10-6*(A, B, D) and *TaGRF11-2A* were major causes for constraining the tillering of *dmc*.

### Expression patterns of *TaGRFs* in response to IAA and GA treatments

**Figure 10:**
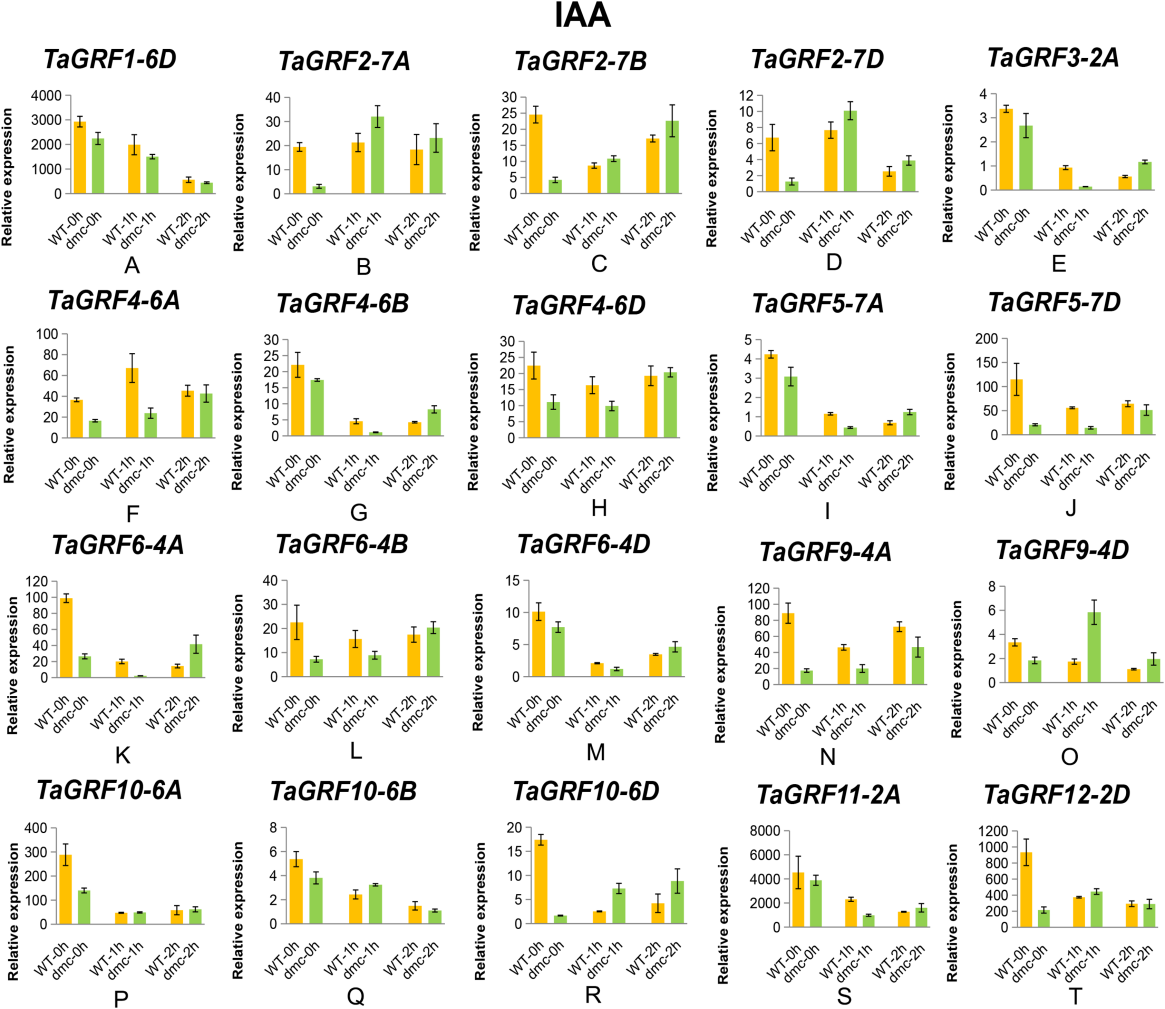
Expression profiles of 20 representative *TaGRF* genes in response to IAA treatment. (A–T) The qRT-PCR results of 20 *TaGRF* genes in response to IAA treatment. Data were normalized to β-actin gene and vertical bars indicated standard deviation.

A total of 20 representative *TaGRFs* were selected from the 30 *TaGRF* genes to investigate whether their expressions were affected by IAA and GA treatments. These *TaGRF* genes were carefully selected based on the *cis*-acting elements in their promoters (Fig. 5) and their expression levels in tillers of *dmc* (Fig. 9). The expressions of 20 *TaGRFs* were affected by IAA treatment in various degrees (Fig. 10). The expressions of 9 *TaGRFs* in WT and 2 *TaGRFs* (*TaGRF1-6D* and *TaGRF10-6B*) in *dmc* were continuously significantly repressed by IAA treatment, their expressions decreased by more than 50%. Among these genes, there was a *cis*-acting element involving in auxin (AuxRR-core, TGA-element) in the 2,000 bp upstream sequences before transcription start positions of *TaGRF1-6D, TaGRF3-2A, TaGRF4-6B, TaGRF6-4A, TaGRF9-4D* and *TaGRF12-2D*. The expression levels of 8 *TaGRFs* in Guomai 301 and 9 *TaGRFs* in *dmc* was down-regulated at 1 hour and up-regulated at 2 h after IAA treatment. There were 2 and 3 *cis*-acting elements involving in auxin in the 2,000 bp upstream sequences before transcription start positions of *TaGRF4-6D* and *TaGRF5-7D*, respectively. Therefore, the expressions of most *TaGRFs* were repressed by IAA at 1 h after treatment. The expression levels of *TaGRF4-6A, TaGRF4-6D, TaGRF6-4B* and *TaGRF10-6A* were almost the same in WT and *dmc* at 2 h after IAA treatment. The expressions of *TaGRF2-7B, TaGRF4-6A, TaGRF6-4B, TaGRF9-4A* and *TaGRF10-6D* in *dmc* were significantly up-regulated by IAA treatment, which suggested these *TaGRFs* played key roles in regulating wheat tillering.

**Figure 11:**
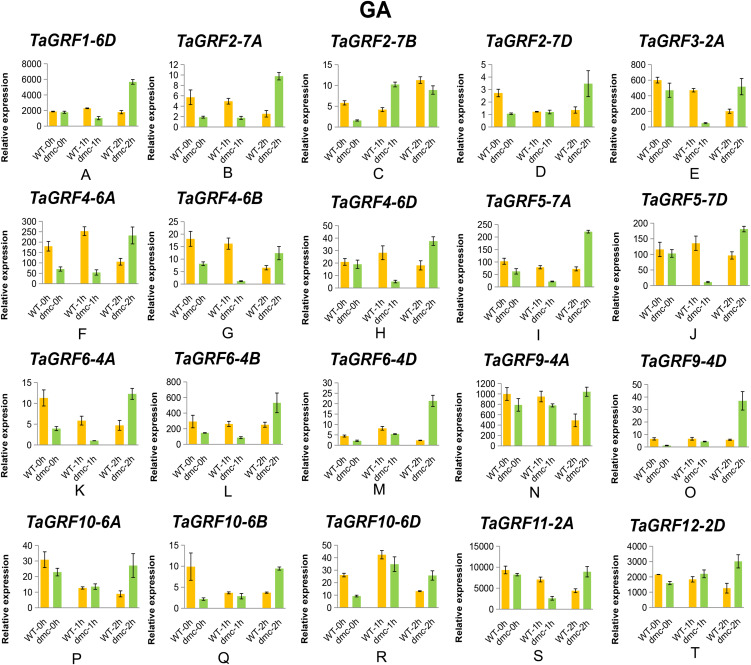
Expression profiles of 20 representative *TaGRF* genes in response to GA treatment. (A–T) The qRT-PCR results of 20 *TaGRF* genes in response to GA treatment. Data were normalized to β-actin gene and vertical bars indicated standard deviation.

The expressions of the 20 *TaGRFs* were also affected by GA treatment in various degrees (Fig. 11). It was worth mentioning that most of the homoeologous genes showed similar expression patterns in response to GA treatment, such as *TaGRF4, TaGRF5, TaGRF6, TaGRF9* and *TaGRF10*. The expressions of *TaGRF1-6D*, *TaGRF6-4B* and *TaGRF9-4D* in WT were hardly affected by GA treatment, while they were significantly up-regulated in *dmc* at 2 h after GA treatment. There were 10 *TaGRFs* in *dmc* were significantly repressed at 1 h and significantly up-regulated at 2 h after GA treatment. The expressions of all the detected *TaGRFs* were significantly up-regulated in *dmc* after GA treatment. There were 1–3 *cis*-acting elements (indicated in the parentheses behind the gene symbols) involving in gibberellin (P-box) in the 2,000 bp upstream sequences before transcription start positions of *TaGRF5-7A* (1), *TaGRF6-4A* (3), *TaGRF6-4B* (2), *TaGRF6-4D* (2), *TaGRF9-4D* (1) and *TaGRF10-6D* (1), respectively. The expressions of all *TaGRFs* were affected by both IAA and GA treatments indicated that phytohormone IAA and GA were involved in regulating wheat tillering.

## Discussion

### Characteristics and evolution of wheat *GRF* gene family members

Plant GRF proteins typically have two conserved domains, QLQ and WRC, in the N-terminal regions. The QLQ domain interacts with GRF-interacting factors (GIFs) to form transcription activating factor that participates in the biological processes of plant growth and development (Kim & Kende, 2004). The WRC domain interacts with the *cis*-acting regions of its downstream genes and plays the biological function of GRF. The genome-wide analyses of *GRF* gene families have been widely carried out in various plant species whose genomes have been sequenced (Cao et al., 2016). In this study, a total of 30 wheat GRF proteins with the two conserved domains, QLQ and WRC, were identified. Phylogenetic and gene structure analyses showed that most of the *GRF* genes in the same subfamily had similar exon/intron structures, which provided clues to the evolutionary relationships of plant *GRFs* (Hu & Liu, 2011). These data indicated that the *GRF* genes with similar structures have similar evolution histories and functions (Babenko et al., 2004; Roy & Penny, 2007). Here, we found wheat homoeologous genes of *TaGRF3-2*(A, B, D) and *TaGRF10-6*(A, B, D) were highly homologous to *OsGRF3* and *OsGRF10*. The distributions of the introns and exons in *TaGRF* genes were similar to those of rice *GRF* genes (Cao et al., 2016), suggesting that *TaGRF* genes and *OsGRF* genes might have similar functions. The gene duplication events are usually derived from the polyploidization or tandem and segmental duplication, segmental duplication occurs most frequently in plants because most plant species are diploidized polyploids (Moore & Purugganan, 2005; Adams & Wendel, 2005). No tandem repeat genes were found in *TaGRFs*, there were 24 homoeologous genes in 27 segmental duplication *GRF* genes, and not every *GRF* had three homoeologous genes on the homoeologous chromosomes A, B and D. It had been reported that some homoeologous genes could be lost during the polyploidization of the genome (Lynch & Force, 2000). Hence, it was highly possible that the segmental duplication played an important role in the expansion of the *TaGRFs*. A large number of *cis*-acting elements related to growth and development, hormones and stress regulation were found in the promoter regions of *TaGRF* genes, which implied their various functions.

### Various functions of *TaGRFs*

Plant *GRFs* regulate growth and development. *AtGRF5* stimulates Arabidopsis chloroplast division, photosynthesis and leaf longevity (Vercruyssen et al., 2015). Overexpression of *ZmGRF10* can reduce leaf size and plant height of maize (Wu et al., 2014). *OsGRF1* not only regulates growth at the juvenile stage, but also regulates heading in rice (Luo et al., 2005). *AtGRF4* and *AtGRF5* are involved in the development of leaf size and leaf senescence in Arabidopsis (Horiguchi, Kim & Tsukaya, 2005; Kim & Lee, 2006). As the target genes of miR396, the expressions of *GRF* genes are post-transcriptionally down-regulated by miR396 (Hewezi & Baum, 2012; Lee & Kim, 2014). There are six *GRF* genes (*AtGRF1*-*AtGRF3* and *AtGRF7*-*AtGRF9*) are regulated by miR396 (miR396a and miR396b) in Arabidopsis (Liu et al., 2009). Down-regulated expressions of *AtGRF* genes by miR396 overexpression resulted in Arabidopsis with enhanced leaf adaxial-abaxial defects, narrower leaves, abnormal development of the pistil and compromises the shoot meristem, etc (Jones-Rhoades, Bartel & Bartel, 2006; Rodriguez et al., 2010, 2015; Wang et al., 2011). Overexpression of Ptc-miR396 from *Populus trichocarpa* in tobacco resulted in altered plant growth and flower development (Baucher et al., 2013). miR396 plays an important role in controlling carpel number and pistil development via regulation of the GRF/GIF complex (Liang et al., 2014). OsmiR396d regulates the expressions of *OsGRF* genes and participates in the regulation of flower organ development in rice (Liu et al., 2014).

The functional diversity of genes can be predicted by their tissue-specific expression patterns. *GRF* genes not only have different distributions and molecular structures, but also have different tissue-specific expression patterns. Studies have shown that *TaGRF* genes are highly expressed in active development tissues or organs, and they are relatively lowly expressed in mature tissues or organs (Choi, Kim & Kende, 2004; Wu, Wang & Zhuang, 2017). *TaGRFs* express highly in young spikes and roots, which is consistent with the putative function based on the meristem-related *cis*-acting elements in the promoter regions of *TaGRF*s. *AtGRF1* and *AtGRF3* are the most highly expressed genes in Arabidopsis roots, which are essential for root development (Szakasits et al., 2009; Hewezi et al., 2012). Overexpression of AtMIR396a decreased the transcript levels of *AtGRF* genes and resulted in a shorter root phenotype (Bao et al., 2014). *TaGRF11* and its homoeologous genes (*TaGRF11-2A, 2B, 2D*) were highly expressed in various tissues, indicating they played essential roles in wheat growth and development (Fig. 8). *TaGRF1-6A, TaGRF6-4A* and their homoeologous genes expressed highly in roots suggesting their functions in root development (Fig. 8). The data indicated that *TaGRFs* functioned differently during wheat development.

### The key *TaGRFs* involved in tiller development

Plant miR396/GRF is a conservative plant growth regulation module, but there is no definite research report on its involvement in plant branching and tillering (Liebsch & Palatnik, 2020). Overexpressions of *OsGRF3* and *OsGRF10* reduce formations of tillers and internodes in rice (Kuijt et al., 2014). Therefore, we speculated that the homologous genes of *TaGRF3* and *TaGRF10* might be related to the growth and development of tiller in wheat. The tiller ability of the transgenic wheat overexpression miR396 is significantly decreased (Song, 2016). Our miRNome and transcriptome integrative analysis about the mutant *dmc* and WT found that the highly expressed tae-miR396b (*T. aestivum* microRNA396b) down-regulated the expressions of many *TaGRFs* (*TaGRF1*, *TaGRF2*, *TaGRF5*, *TaGRF9*, *TaGRF10* and *TaGRF12*) in *dmc* during tillering (He et al., 2018; An et al., 2019). It demonstrated that the miR396/*GRF* regulatory module played a key role in wheat tiller development. Compared with the WT, the expression levels of all *TaGRFs* in *dmc* were significantly decreased at early tillering stage, which is positively related to the phenotype of *dmc* (Fig. 9). All the 30 *TaGRFs* have different expression patterns in WT and *dmc*, but only those significantly differentially expressed *TaGRFs* in TPs are the key tiller development regulators. In this case, *TaGRF1-6D, TaGRF2, TaGRF4, TaGRF5-7A, TaGRF11-2B* and *TaGRF12-2D* were significantly differentially expressed at early tillering stage, indicating their important roles in regulating tiller numbers in wheat. In summary, *TaGRF2-7*(A, B, D), *TaGRF5-7D, TaGRF10-6*(A, B, D) and *TaGRF11-2A* were key regulators in wheat tiller growth and development, especially in *dmc*.

### Exogenous IAA and GA affect the expressions of *TaGRFs*

There is considerable evidence that GRFs play significant roles in regulating plant growth and development, and play a variety of regulatory roles in signal transduction and stress response of plants. There are a large number of *cis*-acting elements related to hormones in *TaGRF* promoters, including those related to IAA (AuxRR-core, TGA-element) and GA (P-box). IAA regulates plant morphological formation, such as plant tropism growth, root development and tiller formation (Li et al., 2000; Ren, Dai & Liu, 2012). OsmiR167a represses its targets *OsARF12* (auxin response factor, ARF), *OsARF17* and *OsARF25*, to control rice tiller angle by fine-tuning auxin asymmetric distribution in shoots (Li et al., 2020). GA participates in regulation of many physiological processes in wheat including the spike development, stem elongation, plant height and stress responses (Zhang et al., 2007; Pavlista, Santra & Baltensperger, 2013). KNOX (KNOTTED1—like homeobox) proteins contribute to the regulation of meristem maintenance by negatively regulating the production of GA (Sakamoto et al., 2001; Bolduc & Hake, 2009). Repression the activities of KNOX genes is a conserved function of *GRFs* (Kuijt et al., 2014). These results suggested that the *GRF* genes positively regulated the production of GAs, and GAs in turn up-regulated *GRF* gene expressions (Wang et al., 2014). *OsGRF1* may play a regulatory role in GA-induced stem elongation (Van der Knaap, Kim & Kende, 2000). GA treatment increases the expressions of some *OsGRFs* (*OsGRF*1, 2, 3, 7, 8, 10 and 12) in rice, while the expression of *OsGRF9* decreases (Bao et al., 2014). Interactions between exogenous cytokinin and nitrogen treatment can regulate tiller bud growth in winter wheat (Yang et al., 2020). There are many evidences of IAA and GA affect tiller developments (Lee, Foster & Morgan, 1998; Frantz et al., 2004; Liu et al., 2011; Assuero et al., 2012; Cai et al., 2013). In previous studies, we found that the contents of IAA were significantly lower, but the contents of GA were significantly higher in the tiller tissues of *dmc* (An et al., 2019). The results of qRT-PCR also confirmed that the expressions of *TaGRFs* were significantly affected by IAA and GA applications (Figs. 10 and 11). According to these data, it was considered that *TaGRFs* as well as IAA and GA signaling were involved in regulating wheat tiller development.

## Conclusions

A total of 30 TaGRF transcription factors with both typical QLQ and WRC conserved domains were identified in wheat. *TaGRF* genes distribute on 12 of the 21 wheat chromosomes, and their promoter regions have a large number of *cis*-acting elements related to plant growth and development, hormone signal pathway and stress response. The expression levels of all the *TaGRF* genes are significantly lower in *dmc* at the early tillering stage (three-leaf stage), and the expressions also are significantly affected by exogenous IAA and GA. *TaGRF1-6A*, *TaGRF2-7D*, *TaGRF9-4A* and *TaGRF11-2A* play basic roles in wheat tiller development. The abnormal expressions of *TaGRF2-7*(A, B, D), *TaGRF5-7D, TaGRF10-6*(A, B, D) and *TaGRF11-2A* are major causes constraining the tillering of *dmc. TaGRFs* and IAA, GA signaling are involved in controlling wheat tillering.

## Supplemental Information

10.7717/peerj.11235/supp-1 Supplemental Information 1 The raw data for qPT-PCR.

10.7717/peerj.11235/supp-2 Supplemental Information 2 List of primers used for *TaGRF* genes expression analysis.

10.7717/peerj.11235/supp-3 Supplemental Information 3 The expression levels of *TaGRF* genes in different tissues of Chinese Spring and Azhurnaya.

10.7717/peerj.11235/supp-4 Supplemental Information 4 GO annotation of *TaGRF* genes.

10.7717/peerj.11235/supp-5 Supplemental Information 5 The expression levels of *TaGRF* genes in WT and dmc.

10.7717/peerj.11235/supp-6 Supplemental Information 6 The numbers of syntenic gene pairs between wheat and Arabidopsis, rice, maize, respectively.

10.7717/peerj.11235/supp-7 Supplemental Information 7 The gene pairs of duplication of *GRF* genes in the wheat genome.

10.7717/peerj.11235/supp-8 Supplemental Information 8 One-to-one orthologous relationships between wheat and wheat, Arabidopsis, rice, maize, respectively.

## Additional Information and Declarations

### DNA Deposition

The following information was supplied regarding the deposition of DNA sequences: Raw sequencing reads are available at NCBI under BioProject ID PRJNA670838.

### Data Availability

The following information was supplied regarding data availability: The raw measurements for qRT-PCR are available in the Supplemental File.
